# Eco-Efficiency and Private Firms’ Relationships with Heterogeneous Public Stakeholders in China

**DOI:** 10.3390/ijerph17196983

**Published:** 2020-09-24

**Authors:** Jiawen Chen, Linlin Liu

**Affiliations:** 1 School of Management, Guangzhou University, Guangzhou 510006, China; jiawenchen@jnu.edu.cn; 2 School of Business Administration, South China University of Technology, Guangzhou 510641, China

**Keywords:** eco-efficiency, stakeholder relationship, stakeholder salience, organizational learning

## Abstract

Private firms have been struggling to simultaneously achieve both environmental and economic goals. The concept of eco-efficiency captures the extent to which firms gain competitiveness through environmental management. Based on stakeholder salience theory and organizational learning theory, this study proposes that relationship with public stakeholders can hinder or promote private firms’ eco-efficiency. Our findings showed that firm eco-efficiency is reduced by a relationship with the government but is enhanced by relationships with non-governmental organizations (NGOs). This study also found that the effects on eco-efficiency of a firm’s relationship with public stakeholders are contingent on firm size. The findings of this study shed light on the organizational learning perspective of eco-efficiency and multi-stakeholder management by theoretically and empirically differentiating the effects on firm eco-efficiency of relationships with the government and NGOs.

## 1. Introduction

With increased global environmental awareness, the attention of business managers has gradually shifted from economic efficiency to eco-efficiency [1,2]. Firm eco-efficiency systematically combines economic and environmental performance [3]. From the organizational learning perspective of firm eco-efficiency, prior studies have suggested that firms engage in an internal learning process to establish the dynamic capability to gain competitive advantage through environmental investment [4,5]. Other scholars have emphasized the knowledge contribution of primary stakeholders to the implementation of solutions that deliver mutual gains of environmental initiative and economic competitiveness [6].

However, extant studies of firm eco-efficiency have neglected the influence of a firms’ relationship with public stakeholders. There is mounting evidence that public stakeholders, such as the state, community groups, and non-governmental organizations (NGOs), are able to induce firms to respond to their needs and claims [7,8]. The importance and heterogeneity of public stakeholders should be stressed in exploring firm eco-efficiency [9]. To fill this research gap, this study explored whether and how a firm’s relationships with heterogeneous public stakeholders affect its eco-efficiency.

Drawing on stakeholder salience theory and organizational learning theory, we aimed to provide a more nuanced conceptualization of the salience of a given public stakeholder relationship in eco-efficiency improvement. While stakeholder salience theory explains how firms allocate attention to different stakeholders’ requests and expectations [10], organizational learning theory helps elucidate the processes that firms search for, assimilate, and transform environmental knowledge for eco-efficiency improvement [11]. Integrating both theoretical perspectives enabled a more comprehensive understanding of a firm’s stakeholder management for efficiency improvement. Previous studies have focused on the extent of stakeholder salience with respect to power, legitimacy, and urgency [10,12], but they have neglected the source of salience for different public stakeholders. In this study, we focused on the sources of salience of the government and NGOs. Two important stakeholders had been emphasized in prior research on corporate environmental responsibility [13] as exerting the dominant influence on firms’ environmental actions. We identified that a relationship with the government is characterized by the power asymmetry of direct power, the conferral of political legitimacy, and the time-sensitivity of environmental concerns; by contrast, a relationship with an NGO gains salience through indirect power over firms, the endorsement of normative legitimacy, and the criticality of environment improvement. Based on these distinctions, we theorized why these different public stakeholder relationships have different effects on firms’ organizational learning for eco-efficiency. Furthermore, previous studies usually treated firm size as a control variable, neglecting its value as a moderator that shapes the influences of public stakeholders on firm eco-efficiency [14]. Firm size is a key condition that either facilitates or inhibiting stakeholder management for eco-efficiency improvement. We thus considered how the effects of relationships with the government and NGOs are contingent on firm size, as firms of different sizes vary in their level of motivation and ability to acquire and leverage external knowledge from stakeholders.

We tested our theory and hypotheses with a sample of private firms in China. Three contributions are offered in this study. First, we theoretically and empirically differentiated the effects on firm eco-efficiency of relationships with two public stakeholders: the government and NGOs. Our findings showed that a firm’s eco-efficiency is negatively associated with the firm’s relationship with the government but positively associated with its relationships with NGOs. Unpacking the heterogeneity of public stakeholder relationships is important to better understand the organizational learning of eco-efficiency improvement and multi-stakeholder management. Second, we went beyond the focus on the extent of stakeholder salience by exploring the source of that salience in multiple stakeholder relationships. We suggest that relationships with public stakeholders differ in the source of salience in terms of power asymmetry, legitimacy conferral, and issue urgency. This elucidates different aspects of stakeholder salience in the theoretical and practical analysis of stakeholder relationship management. Third, we identified how firm size influences the impact of stakeholder relationships on firm eco-efficiency. While prior studies have focused on the environmental management of either large firms or small firms [15], we differentiated and compared the two. Our findings showed that, for large firms, the negative effects of a relationship with the government are stronger and the positive effects of a relationship with an NGO are weaker. This adds more nuance to the process through which firms strategically use their stakeholder relationship to simultaneously achieve eco-efficiency improvement.

## 2. Theory and Hypotheses

### 2.1. Eco-Efficiency

Eco-efficiency refers to a firm’s increased value generation with reduced environmental impact [16]. The logic of eco-efficiency requires a firm to focus on sustainable development, which reflects the need and interests of its stakeholders beyond short-term benefits [17]. To embrace the influence of stakeholders on its goal, a firm must improve its economic performance through proactive environmental management in order to reduce negative impacts on the natural environment and the pollution costs of firm productivity [18]. Thus, the competitive advantages gained through improved eco-efficiency are congruent with the rationale of win–win solutions with stakeholders, which simultaneously create economic and ecological value [3].

To improve its eco-efficiency, a firm must systematically integrate and coordinate knowledge of the environment and market with a view to achieve sustainable development [2]. According to the organizational learning perspective, a firm improves its eco-efficiency through a learning process of searching for, assimilating, and transforming environmental knowledge from different sources [19,20]. Such knowledge includes green technology, recycled materials, and ecological expertise. Since implicit and tacit environmental knowledge are fundamental to improving eco-efficiency [21], current studies have highlighted the importance of a firm’s learning capabilities and the processes adopted to help the firm adapt its existing knowledge base so as to achieve competitive advantage through environmental investment and management [22,23]. Kabongo and Boiral [5] found that dynamic capabilities developed and internalized for an industrial ecological initiative improve performance in transforming a variety of waste materials into value-added products, thus boosting corporate sustainability and market competitiveness. Russo [4] showed that the early adoption of the standardized environmental management system of ISO 14001 facilitates the learning process needed to implement flexible environmental management that lowers production emission. Moreover, firms may attain a higher eco-efficiency through adaptive learning in interorganizational collaborative networks with customers, suppliers, and competitors [6,7]. The learning routines and processes of transferring and combining knowledge both within and across organizations thus support firms’ eco-efficiency achievements, which, in turn, support the integrative rationale of environmental initiatives and competitive advantage.

Though the organizational learning perspective has provided a comprehensive understanding of how firm eco-efficiency can be improved, current studies have at least two limitations. First, they focus on primary stakeholders, neglecting how public stakeholders such as the government and NGOs affect a firm’s learning process of eco-efficiency improvement. Public stakeholders are critical sources of environmental pressure and related ecological knowledge [11]. Different from primary stakeholders such as consumers and suppliers, public stakeholders have a more profound influence in society and have superior knowledge of environmental practices [24]. Second, the different characteristics of stakeholders are not considered in analyzing firms’ learning processes on environmental practices. Extant literature has demonstrated that firms have distinct relationships with stakeholders in terms of power, legitimacy, and urgency, all of which differently influence firms’ strategic response to stakeholders’ environmental concerns [12,25]. Besides the extent of stakeholder salience, its sources profoundly influence firms’ motivation and ability to acquire and absorb external knowledge from stakeholders [26]. From this perspective, a firm’s organizational learning of ecological initiatives can be constrained or improved by its relationships with different stakeholders. Combining research on stakeholder salience theory and organizational learning theory, we aimed to address these gaps by investigating how firm eco-efficiency is influenced by its relationships with heterogeneous public stakeholders.

### 2.2. Heterogeneity in Relationships with Public Stakeholders

Stakeholder theory has long highlighted the heterogeneity among a firm’s stakeholders and its profound influences on the firm’s strategies [27,28,29]. One of the most important dimensions of this heterogeneity is stakeholder salience, which refers to “the degree to which managers give priority to competing stakeholder claims” [10]. The stakeholder salience framework is intensely applied in firms’ decision making with respect to multiple stakeholders, and it suggests that managerial perceptions of stakeholder salience are primarily influenced by three attributes: power, concerning stakeholders’ ability to impose their will on firms [30]; legitimacy, concerning the appropriateness and desirability of stakeholders’ claims [31]; and urgency, concerning the degree to which stakeholders’ claims necessitate immediate attention [10]. Greater stakeholder salience on these three attributes elevates a firm’s awareness of that particular stakeholder’s needs and interests, thus driving the firm’s responsiveness to social and environmental responsibility [11,32].

The sources of salience also differ in a firm’s relationships with multiple stakeholders. Relative power, legitimacy types, and issue urgency are heterogeneous for different stakeholders [26]. Moreover, stakeholders may use different strategies to elevate their salience to firms and compel the adoption of their claims in firms’ social initiatives [33,34]. These influencing strategies of stakeholders produce variation in the content of stakeholder salience [9]. Regarding the two types of public stakeholders on which we focused, prior studies have emphasized the prominent influence of both government and NGOs on a firm’s environmental management [12,35]. The government establishes environmental policies and regulations that govern firms’ environmental investment, while NGOs provide normative incentives for firms’ environmental activities [36]. Applying stakeholder salience theory, a firm’s relationships with public stakeholders are heterogeneous in power asymmetry, legitimacy conferral, and issue urgency. Differences in the sources of stakeholder salience are summarized in Table 1.

Power asymmetry is profound within relationships between private firms and the government. Private firms can establish relationships with the government through personal ties with officials or contractual collaboration [37]. The government has direct power over firms since it controls critical resources upon which firms rely for survival and development [38]. A firm’s resource dependence on the government leaves the former in a low-power position with respect to the latter [39], particularly in emerging economies where the governments extensively intervene in markets [40]. By contrast, in their relationships with private firms, NGOs indirectly exert their power through connections to other powerful authorities such as the media [9]; thus, there is little power asymmetry between firms and NGOs. Resource dependence is also rather mutual in such relationships, as firms rely on NGOs to provide environmental knowledge and NGOs receive philanthropic giving from firms [41]. Achieving the common goals of sustainable development necessitates resource complementarity between firms and NGOs, giving them roughly equivalent power in the collaboration [42].

Legitimacy conferral also differs in firms’ relationships with the government and NGOs. A relationship with the government confers political legitimacy, referring to the state perceptions of the firm’s appropriateness and desirability [43]. Political legitimacy can serve to enable firms to access state resources [44] and provide a buffer against external institutional uncertainty [45]. Politically connected firms will proactively respond to the government’s environmental appeals in order to maintain their political legitimacy [46]. By contrast, firms that establish relationships with NGOs can acquire normative legitimacy, which is derived from the norms and values of society [47]. The endorsement of normative legitimacy supports and promotes firms’ accountability for their behaviors. Since NGOs’ goals are oriented toward improving social welfare, relationships with NGOs can be used to justify firms’ behaviors and to ensure consistency with social norms [48]. Firms also act collaboratively to create greater social value in their relationships with NGOs in order to sustain normative legitimacy in society [49].

Finally, the urgency of environmental issues varies distinctly in terms of time sensitivity and criticality in firms’ relationships with the government and NGOs [50]. In a firm’s relationship with the government, the urgency of environmental issues is rather time-sensitive. Governmental officials face social and political pressure to improve the quality of the natural and ecological environment, since indicators of regional environmental performance are closely linked to officials’ promotion. Therefore, they prompt private firms to take immediate action to reduce negative environmental impacts so as to improve regional environmental performance during officials’ terms. By contrast, firms collaborate with NGOs to search for and develop suitable and appropriate solutions to alleviate environmental concerns [51]. In these relationships, the criticality of environmental initiatives in business sectors is more relevant. Prior studies have suggested that firm managers prioritize environmental responsibility goals in their collaboration with NGOs, regardless of the firms’ initial motives for this collaboration [13,52]. Thus, a firm’s relationships with an NGO will drive it to attach more importance to the NGO’s social and environmental claims, resulting in a higher level of stakeholder criticality.

The above discussions suggest that, with respect to stakeholder salience, a firm’s relationships with the government and NGOs differ regarding power asymmetry, legitimacy conferral, and issue urgency. On this basis, we theorized in this study that a firm’s relationships with the government and NGOs prominently influence the firm’s knowledge integration in organizational learning, thus influencing the firm’s eco-efficiency.

### 2.3. Eco-Efficiency and Relationship with Government

In its relationship with the government, a firm is less powerful, pursues political legitimacy, and faces greater time-sensitivity in addressing environmental issue. The salience of the government in this relationship coerces firms to responsively take immediate environmental action, thus hindering the firm’s learning for eco-efficiency improvement. We expect a firm’s relationship with the government to negatively influence its eco-efficiency.

First, high power asymmetry in this relationship limits a firm’s discretion to learn from the government. Prior studies have demonstrated that an interorganizational relationship characterized by power asymmetry may hinder a firm’s learning process by constraining the efficiency of knowledge transfer [53,54,55]. The limited discretion in a relationship with power asymmetry further deters a firm from establishing a routine structure through which it can implement change in organizational learning [56]. For example, in the context of environmental concerns, the government provides politically connected firms with environmental technological support, which potentially enhances firms’ environmental capability. However, when requested by the government to support environmental improvement, firms are required to implement the technology without customization. Thus, firms have low control over the organizational learning process and necessary changes in learning routines in their relationship with the government, resulting in low efficiency in integrating environmental knowledge.

Second, the political legitimacy conferred by governmental connection reduces a firm’s learning motivation. Since a firm’s market status is protected by this relationship [45], it has less incentive to initiate changes in the process of organizational learning [57]. Regarding eco-efficiency, private firms sustain political legitimacy in their relationship with their government by increasing investment in environmental management while holding the production largely unchanged [58]. For example, private firms may control pollution after, rather than during, the production process, which is cost-inefficient but less disruptive to current production. The decoupling of pollution control and production lowers firms’ incentive to learn environmental management in which economic goals coincide with environmental achievement, thus resulting in a lower level of eco-efficiency.

Third, in the firm–government relationship, the time-sensitivity of addressing environmental concerns disorders the process of organizational learning of enhancing eco-efficiency. Organizational learning involves different stages, and it takes time for the effects of actions to become discernible and conclusions to be drawn about action–outcome linkages [59,60]. Time-sensitivity leaves a limited temporal gap between action and effect, thus impeding organizational learning. The time-sensitivity of environmental issues in a relationship with the government requires private firms to take immediate action to reduce their environmental impact [61]. This short time window makes it very difficult for firms to make necessary changes in routine and structure to integrate environmental knowledge into production [62]. The government’s calls for immediate environmental actions thus hinder firms’ organizational learning of environmental management, which, in turn, lowers their eco-efficiency.

In sum, the power asymmetry, political legitimacy conferral, and issue urgency in a firm’s relationship with the government may hinder a firms from integrating environmental knowledge into the production process, thereby reducing firms’ eco-efficiency. Therefore, we hypothesized that:

**H1:**  *A firm’s relationship with the government is negatively associated with its eco-efficiency*.

### 2.4. Eco-Efficiency and Relationships with NGOs

In its relationships with NGOs, a firm has relatively equal power, is conferred with normative legitimacy, and faces criticality for environmental management. An NGO’s stakeholder salience facilitates a firm’s organizational learning by helping the firm to acquire environmental knowledge and integrate it within the production process. We expect a firm’s relationships with NGOs to enhance its eco-efficiency.

First, power symmetry in a firm’s relationships with NGOs facilitates the transfer and transformation of knowledge from NGOs in the process of organizational learning. Knowledge transfer between organizations is more intense and immediate when they have a mutual resource dependence in their relationships. Mutual resource dependence facilitates the construction of interorganizational routines through which knowledge is exchanged [63]. Balanced power also facilitates focal firms’ integration of environmental knowledge gained from connected stakeholders [39]. Mirvis et al. [64] found that firms gain and absorb tacit knowledge for sustainable development through shared interactions and experiences in collaboration with NGOs. The balanced power in firm–NGO collaborations enables NGOs to provide firms with environmental knowledge and help them to integrate such knowledge in the production process.

Second, normative legitimacy guarantees a firm’s effective integration of knowledge from external sources in its relationships with NGOs. Through collaboration with NGOs, firms initiate changes according to the norms and values of social and environmental responsibility [65]. The literature on cross-sector partnerships suggests that firms proactively seek solutions for addressing negative environmental impacts, aiming to sustain normative legitimacy in collaborations with NGOs [66,67]. The context of a firm–NGO partnership provides opportunities for firms to absorb and infuse the win–win rationale of environmental initiatives [68], thus harnessing NGOs’ expertise to enhance firms’ organizational learning of improving eco-efficiency.

Third, the criticality of environmental concerns in a firm–NGO partnership incentivizes the firm’s organizational learning of improving eco-efficiency. NGOs aim to address environmental issues in a sustainable way, and they persuade firms to adopt effective environmental management practices and promote ecological value [69]. Driven by the criticality of environmental concerns, firms may search more extensively for effective environmental techniques in collaborations with NGOs, using a trial and error approach [70]. Managerial awareness is aroused by the criticality of environmental responsibility [71], with the recognition that a competitive advantage can be gained through proactive environmental investment [72]. Therefore, the salience of the criticality of environmental concerns in the firm–NGO relationship can promote the firm’s alignment of environmental initiative and economic competitiveness, thus shaping organizational learning to deliver superior eco-efficiency.

In sum, power symmetry, normative legitimacy conferral, and the criticality of environmental concerns in the firm–NGO relationship facilitate the firm’s organizational learning in environmental management, thereby increasing the firm’s eco-efficiency. Thus, we hypothesized that:

**H2:**  *A firm’s relationships with NGOs is positively associated with its eco-efficiency*.

### 2.5. Moderating Effects of Firm Size

Prior studies have suggested that large firms and small firms differ in environmental action and management, and they follow different patterns of stakeholder management [73,74]. The wisdom coming from study of large firms cannot always be directly applied to small firms. Therefore, we further theorized that the influence on eco-efficiency of a firm’s relationship with public stakeholders is also affected by firm size. By testing the moderating effects of firm size, we were able to deepen the understanding the importance of organizational contingencies when explaining firm environmental management. From the organizational learning perspective, large firms and small firms differ their level of motivation and ability to acquire and leverage knowledge from stakeholders [75].

Large firms are disadvantaged in organizational learning from stakeholders. This is due to the core rigidity caused by large firms’ inertia that results from the large stock of existing knowledge [76]. Core rigidity lowers the motivation to initiate substantial change to effectively incorporate environmental knowledge from stakeholders in the production process [77]. Moreover, large firms tend to have a long-established and structured production process, which poses a greater challenge to integrating external knowledge of the environment into a settled knowledge system [78]. Consequently, in terms of improving eco-efficiency, the deterring effect of a government relationship would be stronger and the facilitating effect of an NGO relationship would be weaker for large firms.

On the contrary, small firms have more incentive and flexibility to acquire and absorb environmental knowledge in established relationships with stakeholders. Small firms have great motivation to complement their lack of internal knowledge of the environment by interacting and engaging with public stakeholders [79]. Given their shorter product cycle and swift technological development, they are also better equipped and structured to integrate external knowledge [80]. Consequently, small firms can better absorb knowledge of the environment in a relationship with the government, and they can more effectively pursue the win–win outcomes of environmental initiative and economic competitiveness in relationships with NGOs.

In sum, the above arguments suggest that compared to small firms, the impact on larger firms’ organizational learning to improve eco-efficiency is more negative in a relationship with the government and less positive in relationships with NGOs. Therefore, we hypothesized that:

**H3:**  *Firm size strengthens the negative impact of a firm’s relationship with the government on its eco-efficiency*.

**H4:**  *Firm size weakens the positive impact on eco-efficiency of a firm’s relationships with NGOs*.

## 3. Research Method

### 3.1. Data and Sample Selection

We tested our hypotheses with a sample of private firms in China. We obtained our data from the database of 2012 National Survey of Chinese Private Enterprises (NSCPE), which is publicly available. The database had been used on prior studies of private firms in corporate environmental responsibility research [81,82]. The questionnaire design and data collection of the survey were jointly conducted by the United Front Work Department of the Central Committee, the All-China Federation of Industry and Commerce, the State Administration for Industry and Commerce of the People’s Republic of China, and the Private Economy Research Institute of China. Using multistage stratified sampling, the research teams generated a random sample from 31 provinces, municipalities, and autonomous regions in China. Following intensive pre-survey trainings, interviewers visited sampled firms and conducted face-to-face interviews using a standard questionnaire. The survey posed a range of questions covering the size, history, investments, and financial characteristics of private firms, as well as the personal information of owners.

We eliminated 151 sampled firms in the financial industry, such as those of banking and insurance, as they are under different accounting practices. After eliminating another 246 observations with unavailable data on important firm characteristics or investments, our final sample comprised 3701 observations.

### 3.2. Measurement

#### 3.2.1. Dependent Variable

We measured eco-efficiency as the ratio of the value added to environmental impact. This measure has been widely used and validated in prior studies in different industries and countries [1,3]. Valued added captures the value created and retained by a firm, and it is calculated by the firm’s total sales minus the costs of purchased materials and services. For environmental impacts, we used a pollution scale. In China, the government charges firms with an annual fee according to the amount of pollution caused. Thus, the pollution fee indicates a firm’s total negative impact on the environment. Our measure is consistent with the interpretation of eco-efficiency as a firm’s ability to simultaneously minimize pollution and maximize economic performance. The survey asked respondents to report the firm’s total sale, total costs of purchased materials and services, and total amount of pollution fees in 2011. Accordingly, eco-efficiency was operationalized as the difference between total sales and the costs of purchased materials and services divided by the amount of pollution fees.

#### 3.2.2. Independent Variables

Following prior studies [46,58], we measured the relationship with the government using a binary variable. The survey asked the founder “whether you were a party cadre,” whether you were affiliated with the People’s Congress,” and “whether you were a member of the Chinese People’s Political Consultative Conference.” The variable was coded as 1 if the founder reported that he was a party cadre or affiliated with the People’s Congress and/or Chinese People’s Political Consultative Conference, and it was coded as 0 otherwise. The personal ties of private firm founders with the government are ubiquitous and serve as important avenues for political interaction between private firms and the government in China [37].

We used a dummy variable for relationships with NGOs. The survey asked firms whether they formed partnerships with government-affiliated NGOs or independent NGOs: “in the recent two years, whether your company has partnered with NGOs that are initiated by the government” and “in the recent two years, whether your company has partnered with NGOs that are started by other private entities.” Both types of NGOs are legal and legitimate in China because they are officially registered. The variable relationship with NGOs was thus coded 1 when a firm reported having formed partnerships with government-affiliated NGOs or independent NGOs, and it was coded as 0 otherwise.

#### 3.2.3. Control Variables

In addition, we controlled for multiple factors that may influence a firms’ eco-efficiency. For variables concerning firm characteristics, we controlled for firm age and firm size. Younger firms and smaller firms may forge alliances with NGOs to, respectively, mitigate the liability of their newness and smallness [81]. Firm age was calculated as 2012 minus the year when the firm was established. Firm size was measured by total number of employees in the previous year. We also included bank loans that were scaled by sales revenue to control for the firm’s financial leverage. The influence of a firm’s international exposure was also controlled for using firm exports (measured as the total amount of foreign sales income) and by foreign investors’ ownership (measured by the amount of equity owned by foreign investors.

Some of the founders’ demographic factors were also included as control variables. We included the founder’s age, gender, and education to control for founder effects. Founders’ age was measured by the reported age of the founder. Gender was a binary variable and coded 1 if the founder’s reported gender is female. Education was the total number of years of schooling.

Lastly, a set of industrial dummies was included to control for the potential industry-specific effects. We also controlled for market development in the province. Institutional development was measured by institutional indices developed by the National Economic Research Institute (NERI) [83]; these indices indicated the market support institutional infrastructure in the province.

### 3.3. Normality Test

The central limit theorem suggests that when a sample size has over 100 observations, the issue of violation of normality is not a major problem. Though our final sample comprised 3701 observations, the assumption of the normality had to be followed irrespective of the sample size. We checked for the normality of our data using the Shapiro-Wilk test. The Shapiro-Wilk test (*p* = 0.861) was statistically insignificant, implying that the data could be considered normally distributed.

### 3.4. Model Design

An ordinary least-square multiple regression analysis was used to test the association between public stakeholder relationship and firm eco-efficiency. The model of nested regression specifies and accounts for the effect of relationship with the government and NGOs separately. The model is summarized as follows:

Eco-efficiency_i_ = f (relationship with the government, relationships with NGOs, control variables)_i_.

Underlying assumptions of the regressions for multicollinearity were tested using variance inflation factors (VIFs). The VIF values for variables in our models ranged from 1.04 to 1.95, as wis well-below the cut-off of 10 [84]. We thus concluded that there was not serious problem of multicollinearity in our models.

## 4. Results

Table 2 presents the multivariate results of regression analysis for our models. Hypothesis 1 posited the negative effect of relationship with the government. The results from Model 2 provided strong support: eco-efficiency was found to be lower for firms with relationships with the government (β = −0.845; *p* < 0.001). A firm’s established relationship with the government that is characterized by the power asymmetry, political legitimacy conferral, and issue urgency hinders firm eco-efficiency. Hypothesis 1 was thus supported. Hypothesis 2 argued the positive effect of relationships with NGOs. Model 3 showed that the eco-efficiency is higher for firms with relationships with NGOs (β = 0.369; *p* < 0.05). A firm’s established relationship with an NGO that is characterized by power symmetry, normative legitimacy conferral, and the criticality of environmental concerns facilitates a firms’ eco-efficiency improvement. Hypothesis 2 was supported. Therefore, our results reveal the different effects of relationships with two public stakeholders on firm eco-efficiency.

Hypotheses 3 and 4 proposed the moderating role firm size on the association between public stakeholder relationship and eco-efficiency. In Model 5, the interaction between relationship with the government and firm size was found to be significantly negative (β = −0.162; *p* < 0.05). This means that the negative effect of a relationship with the government is stronger for larger firms, a finding that supported Hypothesis 3. Model 6 showed that the coefficient of the interaction between relationships with NGOs and firm size is negative and significant (β = −0.221; *p* < 0.001). This suggested that the positive effect of relationships with NGOs is weaker for larger firms. Thus, Hypothesis 4 was supported. Compared to larger firms, small firms can better utilize the advantages of organizational learning from NGOs and guard against the potential negative influences from the government’s environmental requirement. A summary of the results of hypothesis testing is shown in Table 3.

We conducted a number of additional tests to check for the robustness of the results. First, we used the industry-centered measure of eco-efficiency. Some studies have suggested that industry effects should be taken into consideration when measuring eco-efficiency because co-efficiency values are significantly varied across industries due to different levels of competition [83]. We centered the measure of eco-efficiency on its mean at each industry. The results presented in Table 4 are consistent with our main results. Second, we used the total sale of a firm as an alternative measure of firm size. We took the logarithm of the measure to mitigate potential bias. Models 11 and 12 in Table 5 show that firm size strengthens the negative impact of relationships with the government but weakens the positive impact of relationships with NGOs. Third, we also conducted the subsample regression to further test the moderating effect of firm size on the relationship between public stakeholder relationship and eco-efficiency. We split the sample into small firms and large firms. We used the definition of small firms in prior studies that suggests that small firms are one that has fewer than 50 employees. The split sample results across different firm sizes are reported in Models 13 and 14 in Table 5. The results showed that in the subsample of small firms, the negative effect of a relationship with the government on eco-efficiency is weaker while the positive effect of relationships with NGOs is stronger. Finally, NGOs may differ in term of their monitoring and regulation under governmental agencies, as well as their need to retain favors and credibility with the government. While independent NGOs are strictly regulated and monitored by political agencies to prevent the rise of NGO confrontation with political authorities (Ljubownikow and Crotty, 2014; Spires, 2011), affiliated NGOs can use political connections to avoid such monitoring and promote intimacy with the government (Zheng, Ni, and Crilly, 2019). Therefore, we further investigated the different effects of a firm’s relationships with government-affiliated NGOs and independent NGOs. Model 15 in Table 5 shows that the effects of a firm’s relationship with independent NGOs on firm eco-efficiency are more significant and stronger than from relationships with government-affiliated NGOs. This implies that independent NGOs may be the more suitable partners than government-affiliated NGOs for firms to improve eco-efficiency.

## 5. Discussion

An aim of this study was to examine how a firm’s eco-efficiency is influenced by its relationships with heterogeneous public stakeholders. The issue of eco-efficiency is central to industrial ecology practices that are related to firms’ commitment to environmental initiatives and strategies. Firms are now facing increased environmental pressures and the constraints of resource scarcity. As a result, firms need to develop a deeper understanding of how they are able to more efficiently achieve the environmental goals. Building on prior research indicating that the established relationships with government and NGOs are the main driving forces of firms’ pro-environmental practices [5,26], our study focused on how these public stakeholder relationships lead firms to achieve increased value generation with reduced environmental impact. Interestingly, we found that an improvement of eco-efficiency is hindered by a firm’s relationship with the government because the power asymmetry, political legitimacy conferral, and issue urgency in a firm’s relationship with the government may hinder the process of integrating environmental knowledge into production. However, eco-efficiency improvement was found to be facilitated by a firm’s relationships with NGOs because power symmetry, normative legitimacy conferral, and the criticality of environmental concerns in the firm–NGO relationship facilitate the firm’s organizational learning in environmental management. These findings collaborated with our claims that the influences of public stakeholder relationships on firm eco-efficiency are contingent on salience in those stakeholder relationships. To our knowledge, ours is one of the few studies, if not the only one, that has tested the impact of heterogenous public stakeholder relationships on firm eco-efficiency. The investigation of heterogeneous public stakeholders enables a more comprehensive understanding of a firm’s stakeholder management for efficiency improvement.

Another important issue addressed in this study was how large and small firms may differ in leveraging public stakeholders for eco-efficiency improvement. Large firms are less suited to learning from public stakeholders due to their core rigidity, whereas small firms have high incentive and flexibility to learn from them to compensate for limited internal resources. Therefore, large firms and small firms may differ in utilizing their relationships with public stakeholders for eco-efficiency improvement. As our findings showed that, compared to small firms, the effect on eco-efficiency of large firms is more negative in a relationship with the government and less positive in relationships with NGOs. In other words, small firms are more capable of fending off the government’s disruption on the efficiency of environmental investments and taking advantage of NGOs’ knowledge contribution to advanced eco-efficiency levels. Therefore, firm size is an impactful factor in that it determines a firm’s level of motivation and ability to acquire and leverage external knowledge from stakeholders for environmental management.

## 6. Conclusions

We endeavored to examine how a firm’s relationships with different public stakeholders may affect its eco-efficiency. By applying stakeholder salience theory and an organizational learning perspective to environmental management, we identified the government’s and NGOs’ distinct sources of salience in terms of power asymmetry, legitimacy conferral, and issue urgency, bases upon which we posited that a relationship with the government constrains firms’ eco-efficiency improvement, whereas relationships with NGOs facilitates it. This further implies that the effects of these relationships are contingent on private firm size. Compared to larger firms, small firms can better utilize the advantages of organizational learning from NGOs and guard against the potential negative influences from the government when pursuing eco-efficiency improvement. Our theoretical framework offers numerous future research avenues that may produce a more comprehensive view of stakeholder salience, especially the nuanced interrelationship between stakeholder relationships and firms’ effective environmental management.

We endeavored to make three theoretical contributions. First, we extended research on eco-efficiency by investigating the impacts of relationships with heterogeneous public stakeholders. The concept of eco-efficiency has captured profound theoretical and practical attention in the modern world [2]. Recent studies of eco-efficiency have investigated the internal learning process for resource deployment and orchestration [5], as well as environmental pressure and knowledge provision from primary stakeholders such as customers and suppliers [6,7]. However, the nuanced influences of public stakeholders such as NGOs and the government on firm eco-efficiency are under-researched, although it has been acknowledged that public stakeholders are critical sources of environmental pressure and related ecological knowledge [11]. Following the organizational learning perspective, we demonstrated that a firm’s relationships with salient public stakeholders can also promote or hinder firms’ eco-efficiency. The interactions between a firm and its public stakeholders within such relationships may differently influence the firm’s organizational learning of environmental initiatives. We found that a firm’s eco-efficiency is negatively influenced by a relationship with the government but positively influenced by relationships with NGOs. These findings corroborated with the prior conceptualization of the complex influences (e.g., buffering and enabling) of public stakeholders on corporate environmental investment [12,35]; however, they also transcended this conceptualization by demonstrating that each public stakeholder has a specific impact on firms’ pursuit of win–win solutions, as reflected in eco-efficiency.

Second, we contributed to stakeholder salience theory by exploring the source of salience in multiple stakeholder relationships. Since firms engage in relationships with multiple stakeholders, scholars have suggested that firms respond to stakeholders’ social and environmental claims according to the extent of their salience [10,12]. However, the sources of stakeholder salience in the relationships between firms and their stakeholders have been less discussed in prior research. Majoch et al. [26] found that normative power and organizational legitimacy are the main sources of investors’ salience in driving corporate social responsibility. Extending their research on a single stakeholder, we suggested that the sources of stakeholder salience differ for heterogeneous public stakeholders, with consequential impacts on firms’ environmental management. In a firm’ relationship with the government, stakeholder salience is characterized by direct power asymmetry, the conferral of political legitimacy, and the time-sensitivity of environmental concerns. By contrast, in a firm’s relationships with NGOs, stakeholder salience is characterized by indirect power, the endorsement of normative legitimacy, and the criticality of environmental concerns. Differences in the source of stakeholder salience for the government and NGOs were found to influence firms’ organizational learning of eco-efficiency improvement. Our study offers insight into how sources of salience differ among relationships with heterogeneous stakeholders while also elucidating different aspects of stakeholder salience in the theoretical and practical analysis of multi-stakeholder management.

Third, we expanded research on environmental management by exploring how firm size influences the impacts of stakeholder relationships on firm eco-efficiency. While prior empirical studies of stakeholder management have focused on either large firms [12,13] or small firms [14,39], few studies have contrasted the two. Comparing large and small firms is both theoretically and practically important since they possess different levels of motivation and ability to acquire and absorb external environmental knowledge [73,85]. Our analysis of the contingency of firm size thus improves understanding of how multiple stakeholders influence firm eco-efficiency and adds more nuance to the process through which firms strategically use their stakeholder relationship to simultaneously achieve environmental responsibility and competitive advantage.

Our findings have implications for the environmental management of private firms. A key take away from this study is that the salience of heterogeneous public stakeholders in relationships with firms has different impacts on firms’ learning of how to improve eco-efficiency. This suggests that private firms’ managers should consider stakeholders’ different roles in achieving the dual goals of environmental initiative and competitive advantage. On the one hand, private firms should take measures to vitalize learning incentives and establish a flexible structure to buffer against the negative effect on eco-efficiency of a relationship with the government. On the other hand, private firms can smooth the interaction in relationships with NGOs in order to enhance market competitiveness through effective environmental management.

Our study had several limitations that suggest future research directions. First, given our interests in the different effects of stakeholder relationships on eco-efficiency, we only focused on the government and NGOs as a firm’s critical stakeholders. It would be valuable to try to generalize our theory by investigating stakeholder relationships with other actors, such as business unions and trade associations. Second, due to the limitations of our dataset, we could not directly test the mechanism underlying the impact of stakeholder relationships on firms’ learning for eco-efficiency improvement. Future research could explicitly examine whether different types and processes of organizational learning of environmental management are influenced by stakeholder relationships. Third, our empirical investigation focused on cross-sector partnerships in a single emerging country, which was helpful to control for country-specific heterogeneity. It would be interesting to examine whether our findings hold and how the influences on firm eco-efficiency of a firm’s public stakeholder relationships may vary across different countries. Finally, our study did not consider the difference of organizational culture between the government and NGOs. The organizational cultures of the government and NGOs are important determinants of the establishment of stakeholder relationships and firms’ attitude and response to environmental expectation. Future research can investigate how the organizational cultures of public stakeholders may influence the transfer of environmental knowledge, thus deepening the understanding of firms’ stakeholder management for eco-efficiency improvement.

## Figures and Tables

**Table 1:** Salience in relationships with the government and non-governmental organizations (NGOs).

Salience in Public Stakeholder Relationships	Government	NGO
Power asymmetry	Direct power	Indirect power
Legitimacy conferral	Political legitimacy	Normative legitimacy
Issue urgency	Time-sensitivity	Criticality

**Table 2:** Regression models for firm eco-efficiency.

Variables	Model 1	Model 2	Model 3	Model 4	Model 5	Model 6
Relationship with the government		−0.845 ***		−0.934 ***	−0.876 ***	−0.956 ***
		(0.132)		(0.137)	(0.134)	(0.139)
Relationship with NGO			0.369 *	0.515 ***	0.498 ***	0.585 ***
			(0.145)	(0.148)	(0.149)	(0.160)
Relationship with the government × Firm size					−0.162 *	
					(0.0647)	
Relationship with NGO × Firm size						−0.221 **
						(0.075)
Firm size	0.164 ***	0.258 ***	0.131 **	0.223 ***	0.290 ***	0.308 ***
	(0.039)	(0.042)	(0.039)	(0.041)	(0.063)	(0.052)
Firm age	0.025	0.036 **	0.021	0.032 *	0.030 *	0.031 *
	(0.013)	(0.013)	(0.014)	(0.014)	(0.014)	(0.014)
Leverage	−0.002 *	−0.002 *	−0.002 *	−0.002 *	−0.002 *	−0.002 *
	(0.001)	(0.001)	(0.001)	(0.001)	(0.001)	(0.001)
Export	0.032	0.030	0.031	0.029	0.031	0.034
	(0.026)	(0.026)	(0.026)	(0.026)	(0.026)	(0.026)
Foreign ownership	0.579	0.581	0.607	0.621	0.569	0.630
	(0.754)	(0.745)	(0.766)	(0.760)	(0.757)	(0.752)
Founder age	−0.002	−0.001	−0.001	−0.001	−0.001	−0.001
	(0.007)	(0.007)	(0.007)	(0.007)	(0.007)	(0.007)
Education	−0.034	−0.024	−0.039	−0.029	−0.029	−0.027
	(0.019)	(0.019)	(0.020)	(0.019)	(0.019)	(0.019)
Gender	−0.028	−0.045	−0.028	−0.046	−0.037	−0.032
	(0.163)	(0.163)	(0.163)	(0.162)	(0.162)	(0.162)
Institutional development	0.004	−0.005	0.012	0.004	−0.005	−0.018
	(0.072)	(0.078)	(0.071)	(0.077)	(0.077)	(0.077)
Industry fixed effect	Y	Y	Y	Y	Y	Y
Province fixed effect	Y	Y	Y	Y	Y	Y
_cons	0.432	0.142	0.441	0.124	0.0169	−0.0116
	(0.646)	(0.687)	(0.636)	(0.674)	(0.681)	(0.675)
F value	7.21 ***	6.93 ***	7.18 ***	6.94 ***	6.76 ***	6.75 ***
N	3701	3701	3701	3701	3701	3701

Standard errors in parentheses; F value is the ratio of the mean regression sum of squares divided by the mean error sum of squares; * *p* < 0.05, ** *p* < 0.01, *** *p* < 0.001. Data source: National Survey of Chinese Private Enterprises (NSCPE) database.

**Table 3:** Summary of hypothesis testing.

Hypothesis	Results
**H1:***A firm’s relationship with the government is negatively associated with its eco-efficiency*.	Supported
**H2:***A firm’s relationships with NGOs is positively associated with its eco-efficiency*.	Supported
**H3:***Firm size strengthens the negative impact of a firm’s relationship with the government on its eco-efficiency*.	Supported
**H4:***Firm size weakens the positive impact on eco-efficiency of a firm’s relationships with NGOs*.	Supported

**Table 4:** Robustness check with alternative measure of eco-efficiency.

Variables	Model 7	Model 8	Model 9	Model 10
Relationship with the government	−0.773 ***		−0.820 ***	−0.868 ***
	(0.128)		(0.129)	(0.133)
Relationships with NGOs		0.311 *	0.435 **	0.498 **
		(0.141)	(0.144)	(0.154)
Relationship with the government × Firm size			−0.084 *	
			(0.041)	
Relationships with NGOs × Firm size				−0.172 *
				(0.071)
Firm size	0.129 ***	0.015	0.134 *	0.165 ***
	(0.038)	(0.036)	(0.058)	(0.048)
Firm age	0.038 **	0.025	0.0342 *	0.0346 **
	(0.012)	(0.013)	(0.013)	(0.013)
Leverage	−0.001	−0.001	−0.001	−0.001
	(0.001)	(0.001)	(0.001)	(0.001)
Export	−0.001	−0.004	−0.005	−0.002
	(0.026)	(0.026)	(0.026)	(0.026)
Foreign ownership	0.660	0.682	0.668	0.702
	(0.718)	(0.736)	(0.729)	(0.724)
Founder age	−0.002	−0.002	−0.001	−0.002
	(0.007)	(0.007)	(0.007)	(0.007)
Education	−0.015	−0.028	−0.019	−0.018
	(0.018)	(0.019)	(0.018)	(0.018)
Gender	−0.024	−0.009	−0.021	−0.015
	(0.158)	(0.158)	(0.158)	(0.158)
Institutional development	−0.003	0.011	−0.001	−0.013
	(0.066)	(0.058)	(0.066)	(0.067)
Industry fixed effect	Y	Y	Y	Y
Province fixed effect	Y	Y	Y	Y
_cons	−0.254	0.0193	−0.325	−0.375
	(0.598)	(0.551)	(0.605)	(0.602)
F value	1.73 **	1.74 **	1.99 ***	2.05 ***
N	3701	3701	3701	3701

Standard errors in parentheses; F value is the ratio of the mean regression sum of squares divided by the mean error sum of squares; * *p* < 0.05, ** *p* < 0.01, *** *p* < 0.001. Data source: NSCPE database.

**Table 5:** Robustness check for moderating the effect of firm size.

Variables	Model 11	Model 12	Model 13	Model 14	Model 15
Relationship with the government	−0.831 ***	−0.869 ***	−0.978 ***	−1.142 ***	
	(0.134)	(0.135)	(0.195)	(0.221)	
Relationships with NGOs	0.580 ***	0.642 ***	0.704 *	0.350 *	
	(0.151)	(0.158)	(0.290)	(0.166)	
Relationship with the government × Firm size	−0.041 *				
	(0.022)				
Relationships with NGOs × Firm size		−0.139 **			
		(0.042)			
Relationship with affiliated NGOs					0.211 *
					(0.074)
Relationship with independent NGOs					0.423 **
					(0.113)
Firm size	0.128 ***	0.165 ***	0.072 *	0.097 ***	0.016
	(0.028)	(0.023)	(0.032)	(0.021)	(0.036)
Firm age	0.034 *	0.033 *	0.066 **	−0.006	0.0255
	(0.013)	(0.013)	(0.024)	(0.016)	(0.013)
Leverage	−0.001	−0.001	−0.001	−0.033 *	−0.001
	(0.001	(0.001)	(0.001)	(0.014)	(0.001)
Export	0.041	0.043	−0.041	0.051	−0.004
	(0.026)	(0.026)	(0.058)	(0.029)	(0.026)
Foreign ownership	0.566	0.570	0.903	0.364	0.689
	(0.766)	(0.757)	(1.037)	(0.951)	(0.734)
Founder age	0.001	0.001	−0.011	0.007	−0.002
	(0.007)	(0.007)	(0.012)	(0.008)	(0.007)
Education	−0.027	−0.025	−0.006	−0.062 *	−0.026
	(0.019)	(0.019)	(0.024)	(0.026)	(0.018)
Gender	−0.081	−0.075	−0.315	0.475	−0.009
	(0.160)	(0.160)	(0.197)	(0.306)	(0.158)
Institutional development	−0.025	−0.024	0.067	−0.085	0.011
	(0.072)	(0.075)	(0.043)	(0.111)	(0.058)
Industry fixed effect	Y	Y	Y	Y	Y
Province fixed effect	Y	Y	Y	Y	Y
_cons	0.320	0.0263	0.0176	1.695	0.021
	(0.647)	(0.672)	(0.479)	(1.007)	(0.516)
F value	7.10 ***	7.25 ***	4.10 ***	3.61 ***	1.79 **
N	3701	3701	1866	1835	3701

Standard errors in parentheses; F value is the ratio of the mean regression sum of squares divided by the mean error sum of squares; * *p* < 0.05, ** *p* < 0.01, *** *p* < 0.001. Data source: NSCPE database.
